# Computing Resonant Inelastic X-Ray Scattering Spectra Using The Density Matrix Renormalization Group Method

**DOI:** 10.1038/s41598-018-29218-8

**Published:** 2018-07-23

**Authors:** A. Nocera, U. Kumar, N. Kaushal, G. Alvarez, E. Dagotto, S. Johnston

**Affiliations:** 1 Department of Physics and Astronomy, The University of Tennessee, Knoxville, Tennessee 37996 USA; 2 Materials Science and Technology Division, Oak Ridge National Laboratory, Oak Ridge, Tennessee 37831 USA; 3 Joint Institute for Advanced Materials, The University of Tennessee, Knoxville, TN 37996 USA; 4 Computational Science and Engineering Division and Center for Nanophase Materials Sciences, Oak Ridge National Laboratory, Oak Ridge, Tennessee 37831 USA

## Abstract

We present a method for computing the resonant inelastic x-ray scattering (RIXS) spectra in one-dimensional systems using the density matrix renormalization group (DMRG) method. By using DMRG to address this problem, we shift the computational bottleneck from the memory requirements associated with exact diagonalization (ED) calculations to the computational time associated with the DMRG algorithm. This approach is then used to obtain RIXS spectra on cluster sizes well beyond state-of-the-art ED techniques. Using this new procedure, we compute the low-energy magnetic excitations observed in Cu *L*-edge RIXS for the challenging corner shared CuO_4_ chains, both for large multi-orbital clusters and downfolded *t*-*J* chains. We are able to directly compare results obtained from both models defined in clusters with identical momentum resolution. In the strong coupling limit, we find that the downfolded *t*-*J* model captures the main features of the magnetic excitations probed by RIXS only after a uniform scaling of the spectra is made.

## Introduction

Resonant inelastic x-ray scattering (RIXS) has emerged as a powerful and versatile probe of elementary excitations in quantum materials^1,2^. One of the most commonly used approaches for computing RIXS spectra is small cluster exact diagonalization (ED)^3–21^. This approach is limited by the exponential growth of the Hilbert space, however, which restricts clusters to a relatively small size, thus limiting momentum resolution. For example, ED treatments of multi-orbital spin-chain systems such as the edge-shared CuGeO_3_ or corner shared Sr_2_CuO_3_ have been limited to no more than six CuO_4_ plaquettes^3,6,8,12,22^, while studies carried out using downfolded singleband Hubbard (or *t*-*J*) chains have been limited to ~16–22 sites^4,10,19,20^.

**Figure 1:**
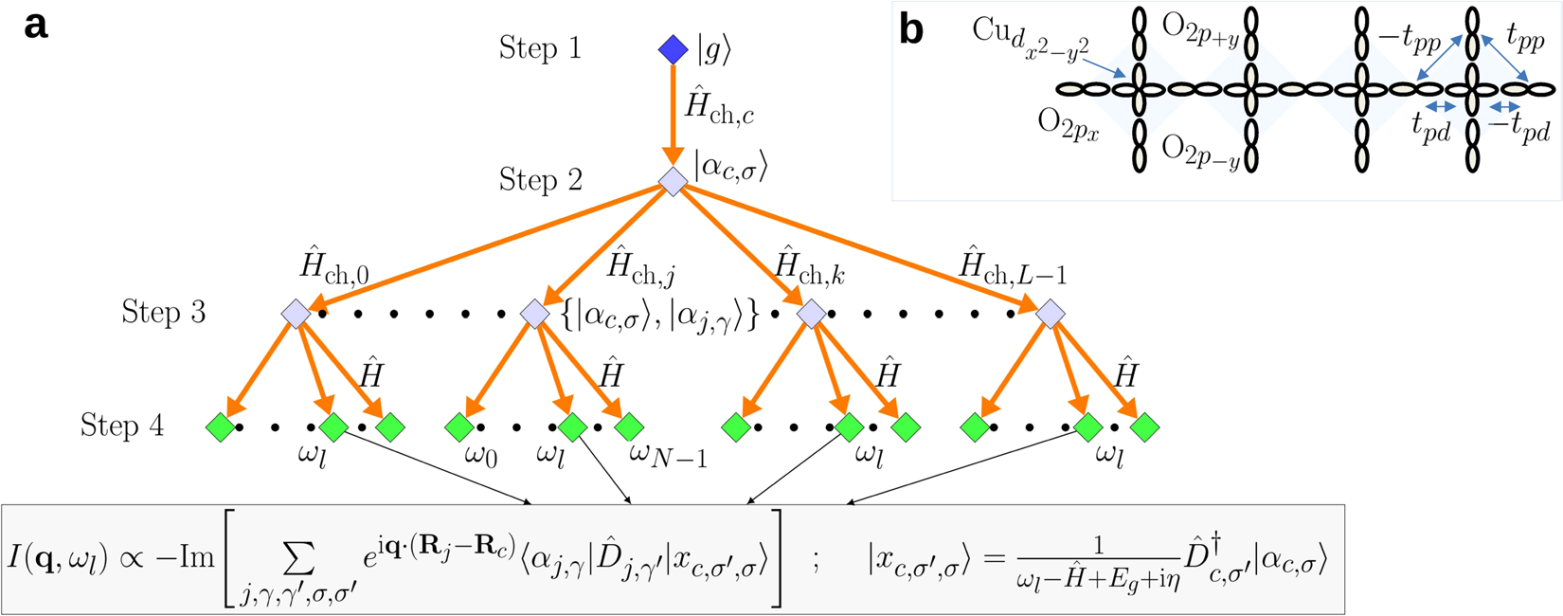
(**a**) A sketch of the algorithm for computing the real space Kramers-Heisenberg formula [Eq. (3)] using the DMRG method at a fixed value of the energy loss Ω = *ω*_*l*_. (**b**) A sketch of the multi-orbital *pd*-model describing the corner shared spin chain cuprates (*e*.*g*. Sr_2_CuO_3_).

The density matrix renormalization group (DMRG) is the most powerful method for computing the ground state properties of strongly correlated materials in one dimension (1D)^23–25^. Within the DMRG framework, several efficient methods are available for computing dynamical correlation functions, including: time-dependent DMRG^26,27^, which computes dynamical correlation functions in the time domain with a subsequent Fourier transform into frequency space^28^; correction-vector methods, which compute the dynamical correlator directly in frequency space^29–32^; continued fraction methods^33–35^; and Chebyshev polynomial expansion methods^36,37^. In this work, we present an efficient algorithm to compute the dynamical correlation function representing the RIXS scattering cross section with DMRG directly in frequency space. We then apply this approach to computing the Cu *L*-edge RIXS spectra of a quasi-1D corner-shared cuprate (*e*.*g*., Sr_2_CuO_3_, see Fig. 1b), a geometry that is challenging for ED calculations due to significant finite size effects^3,6,8^. We consider a multi-orbital Hubbard model that retains the Cu and O orbital degrees of freedom, as well as a downfolded *t*-*J* model. Using our DMRG-based approach, we access systems sizes beyond those accessible to ED, thus enabling us to directly compare the results obtained from the two models on large clusters with comparable momentum resolution.

## The Kramers-Heisenberg Formalism

In a RIXS experiment, photons with energy *ω*_in_ and momentum **k**_in_ (ℏ = 1) scatter inelastically off of a sample, transferring momentum **q** = **k**_out_ − **k**_in_ and energy Ω = *ω*_out_ − *ω*_in_ to its elementary excitations. The resonant nature of the probe arises because *ω*_in_ is tuned to match one of the elemental absorption edges, such that it promotes a core electron to an unoccupied level of the crystal.

The intensity of the RIXS process *I*(**q**, Ω) is given by the Kramers-Heisenberg formalism^1,2^, with

1 $$I({\bf{q}},{\rm{\Omega }})\propto \sum _{f}\,|{F}_{f,g}{|}^{2}\delta ({E}_{f}-{E}_{g}+{\rm{\Omega }}).$$

Here, *E*_*g*_ and *E*_*f*_ are the energies of the ground |*g*〉 and final states |*f*〉 of the system, respectively. The scattering amplitude *F*_*f*,*g*_ is defined as

2 $${F}_{f,g}=\langle f|{\hat{{\mathscr{D}}}}^{\dagger }({{\bf{k}}}_{{\rm{o}}{\rm{u}}{\rm{t}}})\frac{1}{{\omega }_{{\rm{i}}{\rm{n}}}-{\hat{H}}_{{\rm{c}}{\rm{h}}}+{E}_{g}+i{\rm{\Gamma }}}\hat{{\mathscr{D}}}({{\bf{k}}}_{{\rm{i}}{\rm{n}}})|g\rangle ,$$

where $\hat{{\mathscr{D}}}({\bf{k}})$ is the dipole transition operator describing the core-hole excitation. In what follows, we consider the Cu *L*-edge (a Cu 2*p* → 3*d* transition). In this case, the dipole operator is defined as $\hat{{\mathscr{D}}}({\bf{k}})={\sum }_{j,\sigma ,\alpha }\,{e}^{i{\bf{k}}\cdot {{\bf{R}}}_{j}}\times$ $[{A}_{\alpha }^{\hat{\varepsilon }}{\hat{d}}_{j,\sigma }^{\dagger }{\hat{p}}_{j,\alpha ,\sigma }+{\rm{h}}.\,{\rm{c}}.\,]$, where ${d}_{j,\sigma }^{\dagger }$ adds an electron to the valence band orbital $(3{d}_{{x}^{2}-{y}^{2}})$, and ${\hat{p}}_{j,\alpha ,\sigma }$ destroys a spin *σ* electron (creates a hole) in a core 2*p*_*α*_ orbital on site *j* located at **R**_*j*_. The prefactor ${A}_{\alpha }^{\hat{\varepsilon }}$ is the matrix element of the dipole transition between the core 2*p*_*α*_ orbital and the valence $3{d}_{{x}^{2}-{y}^{2}}$ orbital, $\langle 3{d}_{{x}^{2}-{y}^{2},\sigma }|\hat{\varepsilon }\cdot \hat{r}|2{p}_{\alpha ,\sigma }\rangle$, which we set to 1 for simplicity. Γ is the inverse core-hole lifetime, and ${\hat{H}}_{{\rm{ch}}}=\hat{H}+{\hat{H}}^{C}$, where ${\hat{H}}^{C}={V}_{C}\,\sum _{j,\sigma ,\sigma ^{\prime} }\,{\hat{n}}_{j,\sigma }^{d}(1-{\hat{n}}_{j,\sigma ^{\prime} }^{{p}_{\alpha }})$ describes the Coulomb interaction between the core hole and the valence electrons and $\hat{H}$ is the many-body Hamiltonian of the system.

Under the assumption that the core-hole is completely localized, and only one Cu 2*p*_*α*_ orbitals is involved in the RIXS process, Eq. (2) simplifies to

$${F}_{f,g}\propto \sum _{j,\sigma ,{\sigma }^{{\rm{^{\prime} }}}}\,{e}^{i{\bf{q}}\cdot {{\bf{R}}}_{j}}\langle f|{\hat{D}}_{j,\sigma }^{\dagger }\frac{1}{{\omega }_{{\rm{i}}{\rm{n}}}-{\hat{H}}_{{\rm{c}}{\rm{h}},j}+{E}_{g}+i{\rm{\Gamma }}}{\hat{D}}_{j,\sigma ^{\prime} }|g\rangle ,$$

where we have defined the local dipole-transition operator ${\hat{D}}_{j,\sigma }\equiv {\hat{d}}_{j,\sigma }^{\dagger }{\hat{p}}_{j,\sigma }$ and ${\hat{H}}_{{\rm{ch}},j}=\hat{H}+{\hat{H}}_{j}^{C}$, with ${\hat{H}}_{j}^{C}=$ ${V}_{C}\,\sum _{\sigma ,\sigma ^{\prime} }\,{\hat{n}}_{j,\sigma }^{d}(1-{\hat{n}}_{j,\sigma ^{\prime} }^{p})$.

## Reformulation of the Problem for DMRG

The primary difficulty in evaluating Eq. (1) lies in computing the final states |*f*〉. This task is often accomplished using ED on small clusters meant to approximate the infinite system. Obtaining these same final states is usually impossible with DMRG, which *targets* only the ground state; however, we will show that to accomplish this task one can use the Lanczos method, which projects the state onto a Krylov space^38^. Some of the present authors introduced this alternative method to calculate the correction vectors for frequency-dependent correlation functions with DMRG^32^.

We can formulate an efficient DMRG algorithm by expanding the square in Eq. (1), yielding a real space version of the Kramers-Heisenberg formula. To compact the notation, we define vectors $|{\alpha }_{j,\sigma }\rangle \equiv [{\omega }_{{\rm{in}}}-{\hat{H}}_{{\rm{ch}},j}+$ ${E}_{g}+i{\rm{\Gamma }}{]}^{-1}{\hat{D}}_{j,\sigma }|g\rangle$. Using this definition, Eq. (1) can be written as

3 $$I({\bf{q}},{\rm{\Omega }})\propto -\,{\rm{I}}{\rm{m}}\,[\sum _{i,j=0}^{L-1}\,\sum _{\begin{array}{c}\gamma ,\gamma ^{\prime} \\ \sigma ,\sigma ^{\prime} \end{array}}{e}^{i{\bf{q}}\cdot ({{\bf{R}}}_{i}-{{\bf{R}}}_{j})}\langle {\alpha }_{i,\gamma }|{\hat{D}}_{i,\gamma ^{\prime} }\frac{1}{{\rm{\Omega }}-\hat{H}+{E}_{g}+{\rm{i}}\eta }{\hat{D}}_{j,\sigma ^{\prime} }^{\dagger }|{\alpha }_{j,\sigma }\rangle ].$$

Here, *η* is a broadening parameter, which plays the same role as the Gaussian or Lorentzian broadening introduced in ED treatments of the energy-conserving *δ*-function appearing in Eq. (1). Throughout this work, we set it to 75 meV, which is in the range of the typical energy resolution currently attainable in RIXS experiments^39^. Note that the vectors |*α*_*j*,*σ*_〉 must be computed for each value of *ω*_in_ and Γ.

The X-ray absorption spectrum (XAS) can be computed using a similar formalism. Its intensity is given by

4 $$I({\omega }_{{\rm{in}}})\propto -\,{\rm{Im}}\,\sum _{j,\sigma }\,\langle g|{\hat{D}}_{j,\sigma }^{\dagger }\frac{1}{{\omega }_{{\rm{in}}}-{\hat{H}}_{{\rm{ch}},j}+{E}_{g}+i{\rm{\Gamma }}}{\hat{D}}_{j,\sigma }|g\rangle .$$

Finally, we note that we have removed the elastic line from all spectra shown in this work. The precise method for doing this is discussed in Supplementary Note IV.

## Computational Procedure

The algorithm to compute the RIXS spectra using Eq. (3) is as follows (see also Fig. 1a):

- Step 1: Compute the ground state |*g*〉 of $\hat{H}$ using the standard ground state DMRG method. The vector |*g*〉 must be stored for later use.
- Step 2: *Restart* from the ground state calculation, reading and then targeting the ground state vector calculated earlier and using a different Hamiltonian ${\hat{H}}_{{\rm{ch}},c}=\hat{H}+{\hat{H}}_{c}^{C}$, where *j* = *c* is the center site of the chain. Construct the vector |*α*_*c*,*σ*_〉 at the *center* of the chain using the Krylov-space correction vector approach^32^ where we have performed a Lanczos tridiagonalization ${\tilde{T}}_{c}$ with starting vector ${\hat{D}}_{c,\sigma }|g\rangle$, and a subsequent diagonalization ${\tilde{S}}_{c}$ of the Hamiltonian ${\hat{H}}_{{\rm{ch}},c}$, represented in its diagonal form *D*_ch,*c*_ in the Krylov basis. The vector |*α*_*c*,*σ*_〉 should also be stored for later use. Because the cluster is not periodic, the use of a central site here represents an approximation that will become exact in the thermodynamic limit. This central site “trick” was used for the first time in the application of time-dependent DMRG^26^.
  5 $$|{\alpha }_{c,\sigma }\rangle \simeq {\tilde{T}}_{c}^{\dagger }{\tilde{S}}_{c}^{\dagger }\frac{1}{{\omega }_{{\rm{in}}}-{D}_{{\rm{ch}},c}+{E}_{g}+i{\rm{\Gamma }}}{\tilde{S}}_{c}{\tilde{T}}_{c}{\hat{D}}_{c,\sigma }|g\rangle ,$$
- Step 3: *Restart* from previous run, now using a different Hamiltonian ${\hat{H}}_{{\rm{ch}},j}=\hat{H}+{\hat{H}}_{j}^{C}$. Read and then target (in the DMRG sense) the ground state vector |*g*〉, as well as the vector |*α*_*c*,*σ*_〉 constructed in Step 2. For each site *j*, except for the center site considered in Step 2, construct the vector with a Lanczos tridiagonalization ${\tilde{T}}_{j}$ with starting vector ${\hat{D}}_{j,\gamma }|g\rangle$, and a subsequent diagonalization of ${\hat{H}}_{{\rm{ch}},j}$. This step of the algorithm requires a number of runs which is equal to the number of sites minus 1, *i*.*e*., *L* − 1. These can be run in parallel on a standard cluster machine, restarting from Step 2. Performing Step 2 and Step 3 in this sequence is crucial for having the vectors |*α*_*c*,*σ*_〉 and |*α*_*j*,*γ*_〉 in the *same* DMRG basis. The vector |*α*_*j*,*γ*_〉 should also be stored for later use.
  6 $$|{\alpha }_{j,\gamma }\rangle \simeq {\tilde{T}}_{j}^{\dagger }{\tilde{S}}_{j}^{\dagger }\frac{1}{{\omega }_{{\rm{in}}}-{D}_{{\rm{ch}},j}+{E}_{g}+i{\rm{\Gamma }}}{\tilde{S}}_{j}{\tilde{T}}_{j}{\hat{D}}_{j,\gamma }|g\rangle ,$$
- Step 4: *Restart* using the original Hamiltonian $\hat{H}$. Read and then target the ground state vector |*g*〉, the vector |*α*_*c*,*σ*_〉, as well as the vector |*α*_*j*,*γ*_〉 constructed in Step 3. For a fixed Ω = *ω*_*l*_, compute the correction vector of |*α*_*c*,*σ*_〉 using again the Krylov-space correction vector approach as with a Lanczos tridiagonalization $\tilde{T}$ (using ${\hat{D}}_{j,\sigma ^{\prime} }^{\dagger }|{\alpha }_{c,\sigma }\rangle$ as the seed) and a subsequent diagonalization $\tilde{S}$ of the Hamiltonian $\hat{H}$, with *D* being the diagonal form of $\hat{H}$ in the Krylov basis. This is a crucial part of the algorithm, which amounts to computing the correction vector $|{x}_{c,\sigma ^{\prime} ,\sigma }\rangle$ of a previously calculated correction vector |*α*_*c*,*σ*_〉. Execute this computation *N*_Ω_ times for ${\rm{\Omega }}\in [{\omega }_{0},\,{\omega }_{N-1}]$.
  7 $$\begin{array}{ccc}|{x}_{c,\sigma ^{\prime} ,\sigma }\rangle  & \equiv  & \frac{1}{{\rm{\Omega }}-\hat{H}+{E}_{g}+{\rm{i}}\eta }{\hat{D}}_{c,\sigma ^{\prime} }^{\dagger }|{\alpha }_{c,\sigma }\rangle ={\mathop{T}\limits^{ \sim }}^{\dagger }{\mathop{S}\limits^{ \sim }}^{\dagger }\frac{1}{{\rm{\Omega }}-D+{E}_{g}+{\rm{i}}\eta }\mathop{S}\limits^{ \sim }\mathop{T}\limits^{ \sim }\,{\hat{D}}_{c,\sigma ^{\prime} }^{\dagger }|{\alpha }_{c,\sigma }\rangle ,\end{array}$$
- Step 5: Finally, compute the RIXS spectrum in real space ${I}_{j,c}({\rm{\Omega }})\propto \langle {\alpha }_{j,\gamma }|{\hat{D}}_{j,\gamma ^{\prime} }|{x}_{c,\sigma ^{\prime} ,\sigma }\rangle$ (in *I*_*j*,*c*_(Ω) we omit the spin indices *γ*, *γ*′, *σ*, *σ*′ in order to lighten the notation) and then Fourier transform the imaginary part to obtain the RIXS intensity.
  8 $$I({\bf{q}},{\rm{\Omega }})\propto -\,{\rm{Im}}\,\sum _{\begin{array}{c}j,\gamma ,\gamma ^{\prime} \\ \sigma ,\sigma ^{\prime} \end{array}}\,{e}^{i{\bf{q}}\cdot ({{\bf{R}}}_{j}-{{\bf{R}}}_{c})}{I}_{j,c}({\rm{\Omega }}).$$

## Computational Complexity

The computational cost required for DMRG to compute the RIXS spectrum can be easily estimated, assuming that the ground state of the Hamiltonian has already been calculated. Let *C*_2–3_ be the computational cost (*i*.*e*., the number of hours) for a single run in Step 2 (1 run only) or Step 3 (*L* − 1 runs in total). Let *C*_4_ be the computational cost for a single run in Step 4. The total computational time needed to compute the RIXS spectrum is then CPU_cost_ = *C*_2–3_*L* + *C*_4_*LN*_Ω_, where *N*_Ω_ is the number of frequencies needed in a given interval of energy losses. As explained in the previous section, we use a center site “trick” to reduce the computational cost by a factor of the order of *L* (Eqs (3–8)). For the largest system size considered in this work (20 plaquettes in the CuO_4_ multi-orbital model at half-filling, using up to *m* = 1000 DMRG states), the typical values for CPU_cost_ on a single core of a standard computer cluster are: *C*_2–3_ ~ 2 hours, while *C*_4_ ~ 2 − 24 hours. The computational cost *C*_4_ for Step 4 follows the typical performance profile of the Krylov-space approach found in ref.^32^, where less CPU time is needed to compute the spectra at lower energy-losses. We also note that the calculation of each energy loss is trivially parallelizable. From these assumptions, we estimate the proposed method can compute the RIXS spectrum of a cluster as large as Cu_20_O_61_ in less than a day if enough cores are available. In this specific case, one single core run was needed for ground state calculation, 80 single core runs for Steps 2–3, and 800 single core runs for Step 4.

## Numerical Results for the *t*-*J* Model

We first apply our approach to compute the RIXS spectrum of the 1D *t*-*J* model as an effective model for the antiferromagnetic corner-shared spin chain cuprate Sr_2_CuO_3_ (see Methods). Throughout this paper, we adopt open boundary conditions, work at half-filling, and set *t* = 0.3 eV for the nearest neighbor hopping and *J* = 0.25 eV for the antiferromagnetic exchange interaction. These values are typical for Sr_2_CuO_3_^10,20,21,40–46^.

Before scaling up our DMRG calculations to large systems, we benchmarked our method by directly comparing our DMRG results to ED. The results for a *L* = 16 sites *t*-*J* chain are presented in Supplementary Note I. (We provide a similar comparison for a four-plaquette multi-orbital cluster in Supplementary Note II.) Our DMRG approach gives perfect agreement with the ED result for both the XAS and RIXS spectra, for the largest clusters we can access with ED. All of the DRMG simulations presented in this work used up to *m* = 1000 states, with a truncation error smaller than 10^−6^.

**Figure 2:**
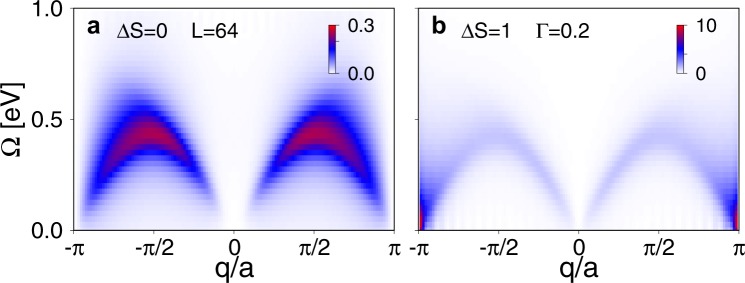
DMRG results for the RIXS intensity *I*(*q*, Ω) of a half-filled *t*-*J* chain. Results are shown for a *L* = 64 site chain, in the (**a**) Δ*S* = 0 and (**b**) Δ*S* = 1 channels. The remaining parameters are *t* = 0.3 eV, *J* = 0.25 eV, *η* = 75 meV and *ω*_in_ = 0.1 eV (which corresponds to the resonance observed in the XAS).

We now turn to results obtained on a *L* = 64 site chain, as shown in Fig. 2. Here, we present results for the spin-conserving (Δ*S* = 0) and non-spin-conserving (Δ*S* = 1) contributions to the total RIXS intensity. The Δ*S* = 0 contribution corresponds to the *σ* = *σ*′ and *γ* = *γ*′ terms in the Kramers-Heisenberg formula Eq. (3). In this case, only two configurations ($\gamma =\gamma ^{\prime} =\sigma =\sigma ^{\prime} =\uparrow$ and $\gamma =\gamma ^{\prime} =\downarrow$, $\sigma =\sigma ^{\prime} =\uparrow$) have to be explicitly calculated with DMRG, as the other two possible spin-conserving configurations contribute equally by symmetry. The remaining terms with *σ* ≠ *σ*′ and *γ* ≠ *γ*′ determine the non-spin-conserving Δ*S* = 1 contributions to the spectrum. In this case, only one configuration ($\sigma ^{\prime} =\downarrow$, $\sigma =\uparrow$, $\gamma ^{\prime} =\downarrow$, $\gamma =\uparrow$) has been simulated with DMRG, as the *flipped* configuration ($\sigma ^{\prime} =\uparrow$, $\sigma =\downarrow$, $\gamma ^{\prime} =\uparrow$, $\gamma =\downarrow$) contributes equally by symmetry. The remaining two possible non-spin-conserving configurations also give zero contribution to the RIXS spectrum by symmetry.

In Fig. 2, the Δ*S* = 1 part of the RIXS spectrum shows a continuum of excitations resembling the two spinon continuum commonly observed in the dynamical spin structure factor *S*(*q*, *ω*) of one-dimensional spin-1/2 antiferromagnets^47–50^. The Δ*S* = 0 contribution in Fig. 2a shows two broad arcs with maxima at *q* = *π*/2*a*. Notice also the perfect cancellation of the RIXS signal at the zone boundary, which is $q=\frac{\pi }{a}\frac{L}{L+1}$ in open boundary conditions. Our results agree with the ED results of refs^4^,^19^, but with much better momentum resolution. We find that the finite size effects of the magnetic excitations in the *t*-*J* model are mild; we observe only small differences between results obtained on *L* = 16 (shown in Supplementary Note I) and *L* = 64 site clusters.

## Magnetic Excitations in the Multi-orbital *pd*-Model

In the strong coupling limit, the low-energy magnetic response of the spin-chain cuprates are believed to be effectively described by a single orbital Hubbard or *t*-*J* model^51,52^. According to this picture, *holes* predominantly occupy the Cu orbitals at half-filling, while the oxygens along the Cu-Cu direction provide a pathway for superexchange interactions between the nearest-neighbor Cu orbitals. Since our DMRG approach provides access to large cluster sizes, we now compute the RIXS spectrum of a more realistic multi-orbital model. Here, we consider the challenging corner-shared geometry, which suffers from slow convergence in the cluster size. To address this, we consider finite 1D Cu_*n*_O_3*n*+1_ clusters, with open boundary conditions, as illustrated in Fig. 1b for the *n* = 4 case. The Hamiltonian is given in the Methods section. We evaluated the Cu *L*-edge RIXS intensity for this model as a function of *n* for up to *n* = 20 CuO_4_ plaquettes.

**Figure 3:**
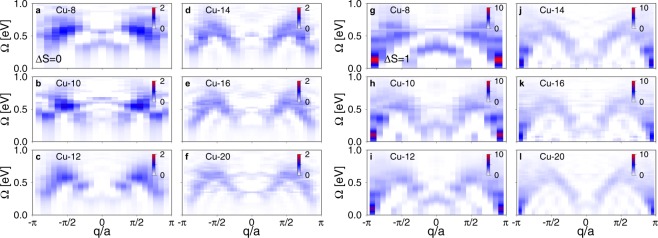
The response *I*(*q*, Ω) obtained with DMRG for a multi-orbital *pd* model as a function of the number of CuO_4_ plaquettes. Panels (a–f) show the spin-conserving Δ*S* = 0 channel of the RIXS intensity, while panels (g–l) show the non-spin-conserving Δ*S* = 1 channel. Results are shown for 8 to 20 unit cells at half filling, computed at resonance with *ω*_in_ = 2.5, Γ = 0.2, and *V*_*C*_ = 4.0 (in eV units).

The RIXS spectra for spin-conserving (Δ*S* = 0) and non-spin-conserving contributions (Δ*S* = 1) calculated with our DMRG method are shown in Fig. 3. Similar to the *t* − *J* spectra, panels (a–f) in Fig. 3 show two broad arcs with maxima at ±*π*/2*a*. Here, we observe significant finite size effects in the RIXS spectra. Some of these effects are the result of our use of the “center-site approximation” in evaluating the Kramers-Heisenberg formula. For example, the downward dispersing low-energy peak centered at *q* = 0 seen in the smaller clusters is the result of this approximation. These features in the spectra can be minimized by carrying out calculations on larger clusters. Because of this, to observe well defined spectral features, we need to consider at least fourteen plaquettes. The *pd* model also shows that the low-energy Δ*S* = 1 part of the RIXS spectrum is characterized by a two-spinon-like continuum of excitations (panels (g–l) in Fig. 3).

## Comparing the Multi-Orbital and Effective *t*-*J* Models

Over the past decade, there has been a considerable research effort dedicated to quantitatively understand the intensity of magnetic excitations probed by inelastic neutron scattering (INS)^44,49,53^. This effort is motivated by the desire to understand the relationship between the spectral weight of the dynamical spin response *S*(**q**, *ω*) and the superconducting transition temperature T_*c*_ of unconventional superconductors^54^. To this end, several studies have set out to determine whether the observed INS intensity can be accounted for by the Heisenberg model in low-dimensional strongly correlated cuprates. In this context, the highest degree of success has been achieved in quasi-1D materials, where accurate theoretical predictions for *S*(*q*, *ω*) are available^44,49^. Many of these studies find that the low-energy Heisenberg model can indeed account for the INS intensity, after including corrections due to effects such as the degree of covalency, its impact on the form factor, and Debye-Waller factors.

RIXS has also been applied to study magnetic excitations in many of the same materials^10,43,46^. It is therefore natural to ponder how covalency modifies the magnetic excitations as viewed by RIXS. In the limit of a short core-hole lifetime, or under constraints in the incoming and outgoing photon polarization, the RIXS intensity for single orbital Hubbard and *t*-*J* chains is well approximated by *S*(*q*, *ω*)^1,5,19,46^. However, to the best of our knowledge, no systematic comparison of the RIXS intensity, as computed by the Kramers-Heisenberg formalism, has been carried out for multi-orbital and downfolded Hamiltonians.

**Figure 4:**
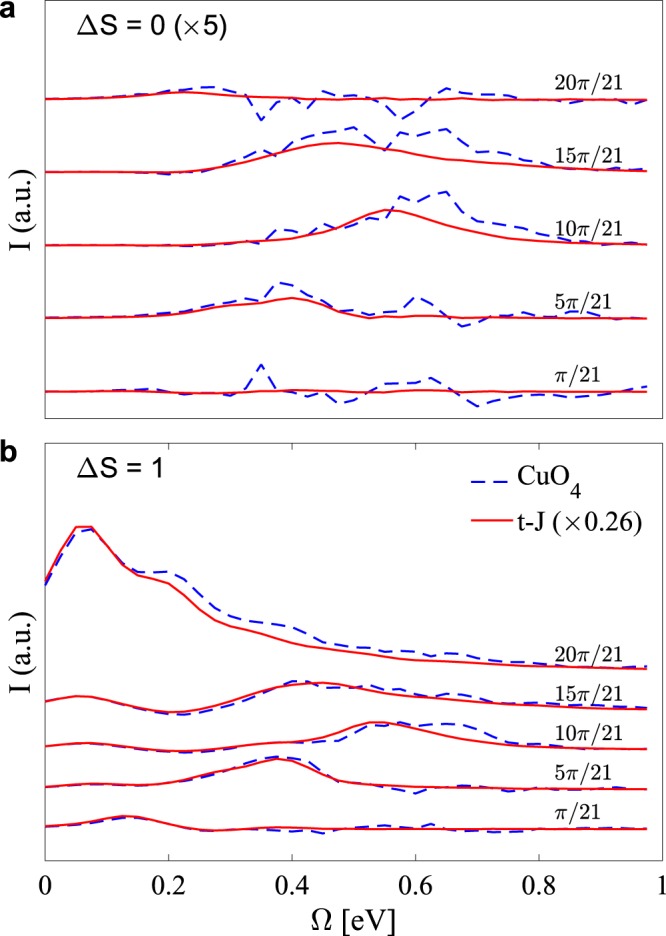
A comparison of the magnetic RIXS excitations computed using DMRG for a 20-site *t*-*J* chain (solid red line) and multi-orbital *pd* model (dashed blue line) with twenty unit cells at half filling. Results are shown for the (**a**) Δ*S* = 0 and (**b**) Δ*S* = 1 channels. The parameters for the *t*-*J* model are *t* = 0.5 eV, *J* = 0.325 eV, *V*_*C*_ = 4 eV, Γ = 0.2 eV, and *ω*_in_ = 0.14 eV. The parameters for the multi-orbital model are given in the main text. The incident photon energy is *ω*_in_ = 2.5 eV, the inverse core hole lifetime is Γ = 0.2 eV, and the core hole potential is *V*_*C*_ = 4 eV. The results for the *t*-*J* model have been scaled by a factor of 0.26 such that the maximum intensity of the Δ*S* = 1 excitations are the same at the zone boundary.

Figure 3 demonstrated that DMRG grants access to large system sizes. We are, therefore, in a position to make such a comparison for the multi-orbital spin-chain cuprates. Figure 4 compares the spectra computed on a *L* = 20 site *t*-*J* chain against those computed on a Cu_20_O_61_ cluster, such that the momentum resolution of the two clusters is the same. The parameters for the multi-orbital model are identical to those used in Fig. 3. To facilitate a meaningful comparison with the *t*-*J* model, we adopted *t* = 0.5 eV and *J* = 0.325 eV. These values are obtained by diagonalizing a Cu_2_O_7_ cluster (see methods). Note that we use the same value of the core hole potential *V*_*C*_ = 4 eV in both cases. In Supplementary Note III, we show results for a reduced value of *V*_*C*_ for the *t*-*J* model, which are very similar. To compare the two spectra, the results for the *t*-*J* model have been scaled by a factor of 0.26 such that the maximum intensity of the Δ*S* = 1 excitations is the same at the zone boundary. This factor presumably accounts for covalent factors and differences in how the core-hole interacts with the distribution of electrons in the intermediate state.

After we have rescaled the spectra, we find excellent overall agreement between the two calculations: the amplitude of the broad arcs for the magnetic excitations, both in the Δ*S* = 0 and in the Δ*S* = 1 channels of the RIXS spectra are well captured by the *t*-*J* model. There are, however, minor quantitative differences related to the spectral weight of the excitations appearing near *q* = *π*/2*a* in the Δ*S* = 0 channel. In this channel, it is natural that the CuO_4_ spectrum is more noisy than the *t* − *J* because we are using the same number of DMRG states for both models, but the Hilbert space of the 20-site CuO_4_ cluster is much larger than the 20-site *t* − *J* cluster. Overall, the *t*-*J* model concentrates the magnetic excitations at slightly lower values of the energy loss in the Δ*S* = 0 channel. This discrepancy might be compensated for by taking a different value of *J*; however, this would come at the expense of the agreement in the Δ*S* = 1 channel. These differences should be kept in mind when one calculates the low-energy magnetic RIXS spectra using an effective *t*-*J* or single-band Hubbard model. Nevertheless, our results suggest that in the strong coupling limit, the magnetic RIXS spectrum can be described qualitatively by the effective *t*-*J* model.

Figure 4 shows that the overall agreement between the full multi-orbital model and the *t*-*J* model is much better in the Δ*S* = 1 channel than in the Δ*S* = 0 channel. We have verified that this discrepancy is not linked to loss of accuracy in our computational algorithm in the Δ*S* = 0 channel. Rather, we can naively understand this difference by recalling the role of charge fluctuations in the two magnetic excitation pathways. The Δ*S* = 1 RIXS excitations are possible in a system with strong spin-orbit coupling in the Cu 2*p* orbitals, which allows the spin of the core-hole to flip in the intermediate state of the RIXS process^4,10,19^. The Δ*S* = 0 pathway, however, requires a double spin-flip between neighboring Cu spins in the final state^4,19^. At the Cu *L*-edge, such processes occur due to charge fluctuations between the neighboring Cu sites in the intermediate state. The multi-orbital model treats such charge fluctuations differently owing to the presence of the ligand oxygen orbitals. This difference accounts for the discrepancy between the two models in the Δ*S* = 0 channel. At the Cu *L*-edge, however, the strong core-hole potential suppresses this difference by repelling holes from the site where it was created resulting in only minor differences between the predictions of the two models.

## Concluding Remarks

We have presented a novel DMRG approach to computing the RIXS spectra and benchmarked this method against traditional ED. Using our DMRG algorithm, we can compute the RIXS spectra on 1D clusters much larger than those accessible to state-of-the-art ED methods. Using the proposed technique, we modeled the magnetic excitations probed by RIXS at the Cu *L*-edge in 1D antiferromagnets on the largest cluster sizes to date. We found that both the full multi-orbital cluster and the effective *t*-*J* model provide comparable descriptions of the magnetic excitations in the Δ*S* = 1 channel, while there were slight differences in the Δ*S* = 0 channel. This discrepancy was explained by noting the different ways that magnetic excitations are created in these channels.

Finally, we note that the bottleneck to RIXS simulations using ED is the exponential growth of the Hilbert space. Our approach shifts the computational burden to the availability of CPUs, thus opening the door to calculations for much larger systems. For example, one can envision extending this approach to the 2D models currently under active study by the DMRG community. Indeed, our RIXS-DMRG method is not restricted to 1D systems and can be applied to a 2D lattice geometry in the same sense specified in ref.^55^. One can always map a finite 2D *N* × *N* lattice with short-range interactions and hoppings into a 1D lattice with *N*^2^ sites and long-range interactions and hoppings. Once such a mapping is obtained, for instance, by drawing a 1D path that scans through the lattice following a “snake”-like pattern, one can straightforwardly apply the conventional DMRG algorithm, and similarly our RIXS-DMRG approach. It is well known, however, that there is a cost to this simplification. Since the interactions have long-range character, the numerical simulations become computationally more expensive as more DMRG states are needed to achieve converged results (area-law entanglement growth). Instead, DMRG applications to 2D systems usually adopt a mapping to multi-leg cylinders^55^. These lattice structures consist of coupled 1D chains or “legs” (usually up to 12 legs for spin systems^55^, and up to 4–6 legs for fermionic systems^56–58^). DMRG simulations are then performed using periodic boundary conditions along the short (“y”) direction and open boundary conditions along the long (“x”) direction. The area-law entanglement growth is still the main limitation of the DMRG algorithm in this case as the number of legs of the cylinder increases^56–58^. The computation of dynamical response functions within the DMRG framework on multi-leg cylinders is computationally even more challenging, but it has been shown to be feasible. Indeed, the computation of spin (*S*(**q**, *ω*)) and charge (*N*(**q**, *ω*)) dynamical response functions of 4-leg *t*-*t*′-*J* cylinders has recently appeared in ref.^59^. Our RIXS-DMRG algorithm can be carried out on similar multi-leg cylinders.

## Methods

The multi-orbital *pd*-Hamiltonian describing the corner-shared spin-chains, given in the hole-picture, is

9 $$\begin{array}{ccc}H & = & {\varepsilon }_{d}\,\sum _{i,\sigma }\,{n}_{i,\sigma }^{d}+\sum _{j,\sigma }\,{\varepsilon }_{p,\gamma }{n}_{j,\gamma ,\sigma }^{p}+\sum _{\begin{array}{c}\langle i,j\rangle \\ \gamma ,\sigma \end{array}}\,{t}_{pd}^{ij}({d}_{i,\sigma }^{\dagger }{p}_{j,\gamma ,\sigma }+{\rm{h}}.\,{\rm{c}}.\,)+\sum _{\begin{array}{c}\langle j,j^{\prime} \rangle \\ \gamma ,\gamma ^{\prime} ,\sigma \end{array}}\,{t}_{pp}^{jj^{\prime} }{p}_{j,\gamma ,\sigma }^{\dagger }{p}_{j^{\prime} ,\gamma ^{\prime} \sigma }\\  &  & +{U}_{d}\,\sum _{i}\,{n}_{i,\uparrow }^{d}{n}_{i,\downarrow }^{d}+{U}_{p}\,\sum _{i,\gamma }\,{n}_{j,\gamma ,\uparrow }^{p}{n}_{j,\gamma ,\downarrow }^{p}+{U}_{pd}\,\sum _{\begin{array}{c}\langle i,j\rangle \\ \sigma ,\sigma ^{\prime} \end{array}}\,{n}_{i,\sigma }^{d}{n}_{j,\gamma ,\sigma ^{\prime} }^{p}.\end{array}$$

Here, $\langle \ldots \rangle$ denotes a sum over nearest neighbor orbitals; ${d}_{i,\sigma }^{\dagger }$ (${p}_{j,\gamma ,\sigma }^{\dagger }$) creates a spin *σ* hole on the *i*^th^ Cu 3${d}_{{x}^{2}-{y}^{2}}$ orbital (the *j*^th^ O 2*p*_*γ*_ orbital, *γ* = *x*, ±*y*); *ε*_*d*_ and *ε*_*p*,*γ*_ are the on-site energies; ${n}_{i,\sigma }^{d}$ (${n}_{j,\gamma ,\sigma }^{p}$) is the number operator for the Cu 3${d}_{{x}^{2}-{y}^{2}}$ orbital (the *j*^th^ O 2*p*_*γ*_ orbital); ${t}_{pd}^{ij}$ and ${t}_{pp}^{jj^{\prime} }$ are the Cu-O and O-O overlap integrals, respectively (the *ij* and *jj*′ dependence only indicates the ± differences among hoppings, Fig. 1b); *U*_*d*_ and *U*_*p*_ are the onsite Hubbard repulsions of the Cu and O orbitals, respectively, and *U*_*pd*_ is the nearest-neighbor Cu-O Hubbard repulsion. The phase convention for the overlap integrals is shown in Fig. 1b. In this work, we adopt (in units of eV) *ε*_*d*_ = 0, *ε*_*p*,*x*_ = 3, *ε*_*p*,*y*_ = 3.5, |*t*_(*p*,*x*)*d*_| = 1.5 |*t*_(*p*,*y*)*d*_| = 1.8, |*t*_*pp*_| = 0.75, *U*_*d*_ = 8, *U*_*p*_ = 4, and *U*_*pd*_ = 1, following ref.^21^.

In the limit of large *U*_*d*_, one integrates out the oxygen degrees of freedom and maps Eq. (9) onto an effective spin-1/2 *t*-*J* Hamiltonian^52^

$$H=-\,t\,\sum _{i,\sigma }\,({\tilde{d}}_{i,\sigma }^{\dagger }{\tilde{d}}_{i+1,\sigma }+h.\,c.\,)+J(\sum _{i}\,{{\bf{S}}}_{i}\cdot {{\bf{S}}}_{i+1}-\frac{1}{4}{n}_{i}{n}_{i+1}).$$

Here, ${\tilde{d}}_{i,\sigma }$ is the annihilation operator for a hole with spin *σ* at site *i*, under the constraint of no double occupancy, ${n}_{i}={\sum }_{\sigma }\,{n}_{i,\sigma }$ is the number operator, and **S**_*i*_ is the spin operator at site *i*.

To facilitate a direct comparison between the two models, one can extract the hopping *t* and exchange interaction *J* from an ED calculation of a two-plaquette Cu_2_O_7_ cluster with open boundary conditions^60^. Here, we obtain the hopping (*t* = 0.5 eV) by diagonalizing this cluster in the ($2\,\uparrow \,,\,1\,\downarrow$)-hole sector, and setting 2*t* to be equal to the energy separation between the bonding and antibonding states of the Zhang-Rice singlet. Similarly, we can obtain the superexchange (*J* = 0.325 eV) by diagonalizing the cluster in the $(1\,\uparrow \,,\,1\,\downarrow \,)$-hole sector, and setting the singlet-triplet splitting of the Cu (*d*^9^*d*^9^) configurations equal to *J*.

### Data and Code availability

The data that support the findings of this study are available from the corresponding author upon request. In the Supplementary Note V we provide details about the code used to obtain the DMRG results.

## Electronic supplementary material

Supplementary Material
