# Supplementary material for: Computing Resonant Inelastic X-Ray Scattering Spectra Using The Density Matrix Renormalization Group Method
Source: Sci Rep. 2018 Jul 23;8:11080. doi: 10.1038/s41598-018-29218-8 (PMC6056525; doi:10.1038/s41598-018-29218-8)
Supplement: Supplementary file 1 — Supplementary Material [file 41598_2018_29218_MOESM1_ESM.pdf]

Computing Resonant Inelastic X-Ray Scattering Spectra Using The Density Matrix Renormalization Group Method

A. Nocera,[1,2,*] U. Kumar,[1,3] N. Kaushal,[1] G. Alvarez,[4] E. Dagotto,[1,2] and S. Johnston[1,3]

[1]*Department of Physics and Astronomy, The University of Tennessee, Knoxville, Tennessee 37996, USA*
[2]*Materials Science and Technology Division, Oak Ridge National Laboratory, Oak Ridge, Tennessee 37831, USA*
[3]*Joint Institute for Advanced Materials, The University of Tennessee, Knoxville, TN 37996, USA*
[4]*Computational Science and Engineering Division and Center for Nanophase Materials Sciences, Oak Ridge National Laboratory, Oak Ridge, Tennessee 37831, USA*

Supplementary Note I: Benchmarks on a 16-site t-J chain

In this note, we compare the results of our DMRG method against the spectrum obtained from Lanczos ED. Supplementary Figure 1 directly compares the results from the two methods applied to a $L = 16$ site t-J chain, where our DMRG approach gives perfect agreement with the ED results for both the XAS and RIXS spectra. Here, we have assumed parameter values typical for a Cu L-edge measurement performed on Sr_2CuO_3 with $t = 0.3$, $J = 0.25$, an inverse core hole lifetime $\Gamma = 0.3$ eV, and a core-hole repulsion $V_C = 2.0$ eV. The two methods give a resonant absorption peak in the XAS for an incident energy $\omega_{\rm in} = 0.1$ eV.

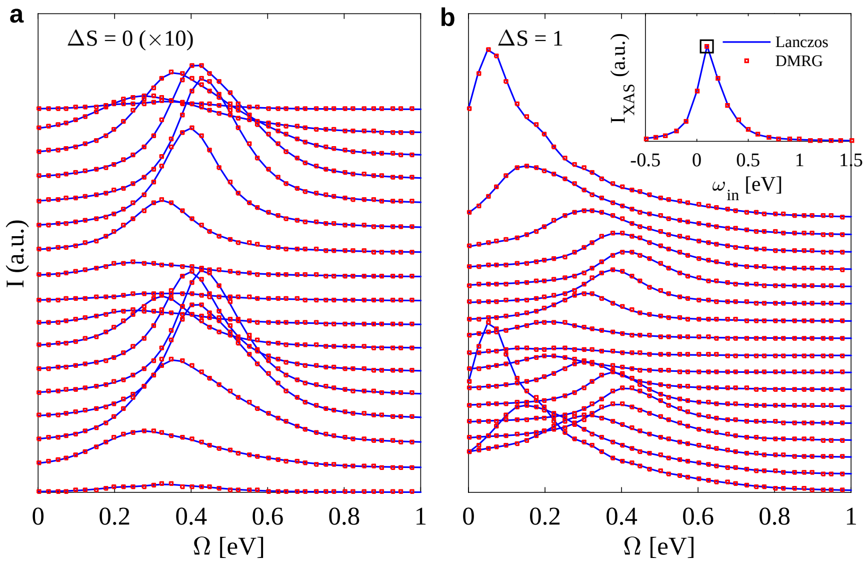

Supplementary Figure 1: A comparison of the RIXS response $I(q,\Omega)$ calculated for a t-J chain of length $L = 16$ at half filling $N = 16$ with $J = 0.25$ eV and $t = 0.3$ eV. Results are shown for both the (a) $\Delta S = 0$ and (b) $\Delta S = 1$ channels, computed at resonance with $\omega_{\rm in} = 0.1$ eV. We have computed the spectrum with DMRG (red squares), which we compare to results obtained from Lanczos ED (solid blue line). The inset of panel (b) compares the XAS spectrum computed with DMRG [using Eq. (8)] and Lanczos ED. The open black box indicates the incident energy $\omega_{\rm in}$ used to compute the RIXS spectra. Note that in this comparison we did not use the center trick for calculating the RIXS spectra. Instead, Eq. (3) of the main text has been used.

Supplementary Note II: Benchmarks on a multi-orbital corner-shared CuO_4 chain

Supplementary Figure 2 presents a second comparison of the results obtained from a multi-orbital Cu_4O_{13} cluster, with open boundary conditions. The model parameters are the same as those used in the main text. Our DMRG approach again gives perfect agreement with the ED result for both the XAS and RIXS spectra.

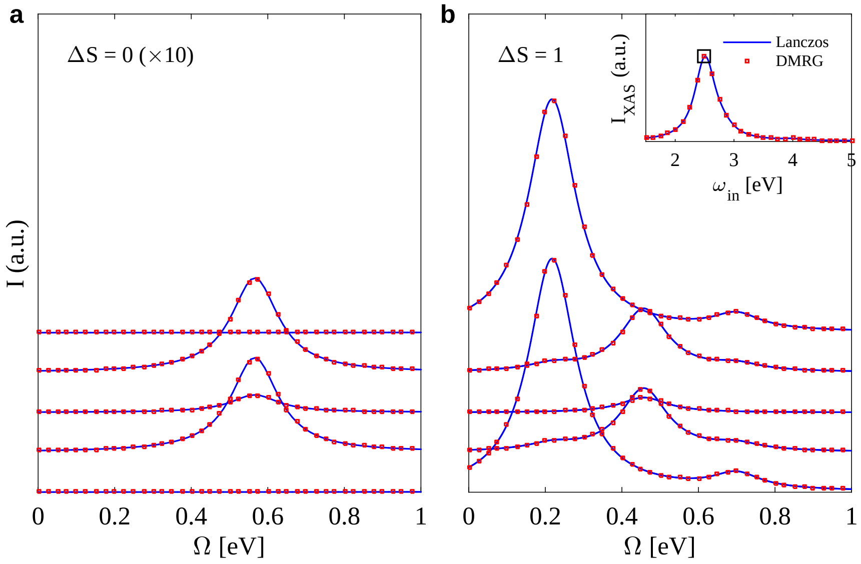

Supplementary Figure 2: A comparison of the RIXS response $I(q,\Omega)$ calculated for a multi-orbital *pd* model for Sr_2CuO_3 with 4 unit cells at half filling $N=4$. The model parameters are identical to those used in the main text. Results are shown for both the (a) $\Delta S=0$ and (b) $\Delta S=1$ channels, computed at resonance with $\omega_{\rm in}=2.5$ eV. The spectrum computed with DMRG (red squares) is compared with results obtained from Lanczos ED (solid blue line). The inset of panel (b) shows a comparison of the XAS spectrum computed with DMRG [using Eq. (8)] and Lanczos ED. The open black box indicates the incident energy $\omega_{\rm in}$ used to compute the RIXS spectra. Note that in this comparison we did not use the center trick for calculating the RIXS spectra. Instead, Eq. (3) of the main text has been used.

Supplementary Note III: Comparison of the Two Models

In the main text, we compared results for the magnetic RIXS spectra of the t-J and multi-orbital pd model, each with 20 unit cells. Here, Supplementary Figure 3 presents a similar comparison but for a different value of the core-hole potential used for the t-J model.

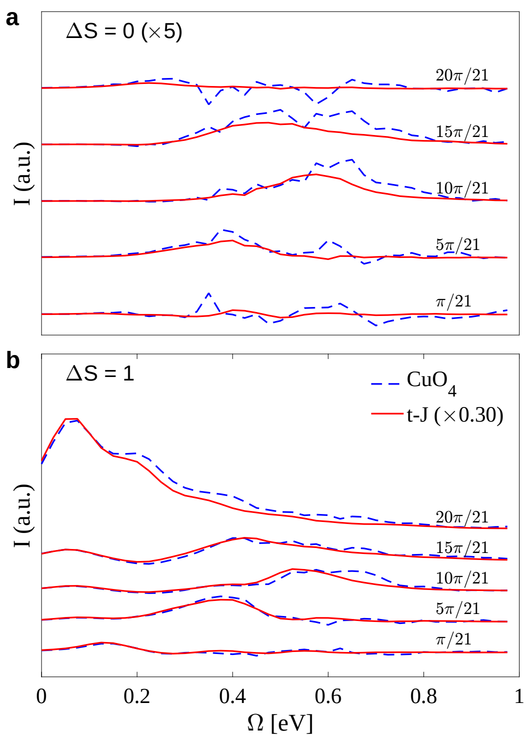

Supplementary Figure 3: A comparison of the magnetic RIXS excitations computed using DMRG for a 20-site t-J chain (solid red line) and multi-orbital pd model (dashed blue line) with twenty unit cells at half filling. Results are shown for the (a) $\Delta S = 0$ and (b) $\Delta S = 1$ channels. The parameters for the t-J model are $t = 0.5$ eV, $J = 0.325$ eV, $\omega_{\text{in}} = 0.04$ eV, and $V_C = 2$ eV. The parameters for the multi-orbital pd model are given in the main text. The incident photon energy is $\omega_{\text{in}} = 2.5$ eV, the inverse core hole lifetime is $\Gamma = 0.2$ eV, and the core hole potential is $V_C = 4$ eV. The results for the t-J model have been scaled by a factor of 0.30 such that the maximum intensity of the $\Delta S = 1$ excitations is the same at the zone boundary.

Supplementary Note IV: Removing the Elastic Line From the DMRG Calculations

We first rewrite Eq. (3) of the main text to explicitly indicate the center site c

$$I(\mathbf{q},\Omega) \propto -\text{Im}\left[\sum_{i=0}^{L-1}\sum_{\substack{\gamma,\gamma'\\ \sigma,\sigma'}} e^{i\mathbf{q}\cdot(\mathbf{R}_i-\mathbf{R}_c)}\langle\alpha_{i,\gamma}|\hat{D}_{i,\gamma'}\frac{1}{\Omega-\hat{H}+E_g+\mathrm{i}\eta}\hat{D}^{\dagger}_{c,\sigma'}|\alpha_{c,\sigma}\rangle\right]. \tag{1}$$

The $\Delta S = 0$ contribution computed with DMRG is then given by the expression

$$I_{\Delta S=0}(\mathbf{q},\Omega) \propto -\text{Im}\left[\sum_{i=0}^{L-1}\sum_{\sigma=\uparrow,\downarrow} e^{i\mathbf{q}\cdot(\mathbf{R}_i-\mathbf{R}_c)}\langle\alpha_{i,\sigma}|\hat{D}_{i,\sigma}\hat{P}\frac{1}{\Omega-\hat{H}+E_g+\mathrm{i}\eta}\hat{P}\hat{D}^{\dagger}_{c,\uparrow}|\alpha_{c,\uparrow}\rangle\right], \tag{2}$$

where $\hat{P} = \mathbb{1} - |g\rangle\langle g|$ projects out the ground-state contribution. The expectation values $\langle \alpha_{i,\sigma}|\hat{D}_{i,\sigma}|g\rangle$ (and their hermitian conjugates) are calculated in Step 3 of the algorithm, and used in Step 4. Here, the contribution to the elastic peak of the spectra is removed by the subtraction of $\langle \alpha_{i,\sigma}|\hat{D}_{i,\sigma}|g\rangle$.

The $\Delta S = 1$ contribution of the RIXS spectrum is given by

$$I_{\Delta S=1}(\mathbf{q},\Omega) \propto -\mathrm{Im}\left[\sum_{i=0}^{L-1} e^{i\mathbf{q}\cdot(\mathbf{R}_i-\mathbf{R}_c)} \langle \alpha_{i,\uparrow}|\hat{D}_{i,\downarrow}\frac{1}{\Omega - \hat{H} + E_g + \mathrm{i}\eta}\hat{D}^{\dagger}_{c,\downarrow}|\alpha_{c,\uparrow}\rangle\right]. \tag{3}$$

In this case, the elastic contribution is absent because $[\hat{H}, S_z^{\mathrm{tot}}] = 0$, thus $\langle \alpha_{i,\sigma}|\hat{D}_{i,\bar{\sigma}}|g\rangle = \langle g|\hat{D}^{\dagger}_{i,\sigma}[\omega_{\mathrm{in}} - \hat{H}_{\mathrm{ch},i} + E_g + \mathrm{i}\Gamma]^{-1}\hat{D}_{i,\bar{\sigma}}|g\rangle = 0$ for $\bar{\sigma} = -\sigma$.

Supplementary Note V: DMRG++

The DMRG++ computer program was used for the DMRG results. DMRG++ is available at `https://github.com/g1257/dmrgpp` under a free and open source license, is maintained, and open for community contributions.

The DMRG++ code can be obtained with:

```
git clone https://github.com/g1257/dmrgpp.git
```

and PsimagLite with:

```
git clone https://github.com/g1257/PsimagLite.git
```

To compile:

```
cd PsimagLite/lib
perl configure.pl
(you may now optionally edit Config.make)
make
cd ../../dmrgpp/src
perl configure.pl
(you may now optionally edit Config.make)
make
```

For the numerical results shown in this manuscript, PsimagLite version 2.02 and DMRG++ version 5.03 were used. Below, we will describe the input files needed to reproduce the data in Fig. 2 of the main text, corresponding to the RIXS spectrum of a $t - J$ chain. For convenience, we include the ground state input file, `inputGS.inp`, valid for a $t - J$ chain with a system size $L = 8$, using only $m = 100$ DMRG states.

```
TotalNumberOfSites=8
NumberOfTerms=4

DegreesOfFreedom=1
GeometryKind=chain
GeometryOptions=ConstantValues
Connectors 1 -0.3

DegreesOfFreedom=1
GeometryKind=chain
GeometryOptions=ConstantValues
Connectors 1 0.25

DegreesOfFreedom=1
GeometryKind=chain
GeometryOptions=ConstantValues
Connectors 1 0.25
```

```
DegreesOfFreedom=1
GeometryKind=chain
GeometryOptions=ConstantValues
Connectors 1 -0.0625

Model=TjMultiOrb

potentialV 16
0 0 0 0 0 0 0 0
0 0 0 0 0 0 0 0

InfiniteLoopKeptStates=100
FiniteLoops 5
 3 100 0
-6 100 0 6 100 0
-6 100 0 6 100 0

TargetElectronsUp=4
TargetElectronsDown=4
Threads=1
SolverOptions=twositedmrg,useSvd
Version=version
OutputFile=dataGS.txt
Orbitals=1
```

This input could be executed quickly on a standard laptop as

```
./dmrg -f inputGS.inp
```

Once the ground state $|g\rangle$ has been obtained, the Step 2 of the algorithm described in Fig. 1 of the main text proceeds computing the vector $|\alpha_{c,\uparrow}\rangle$ for $c = L/2-1$ restarting from the ground state calculations. The input `input_Step2.inp` to accomplish this job is

```
TotalNumberOfSites=8
NumberOfTerms=4

DegreesOfFreedom=1
GeometryKind=chain
GeometryOptions=ConstantValues
Connectors 1 -0.3

DegreesOfFreedom=1
GeometryKind=chain
GeometryOptions=ConstantValues
Connectors 1 0.25

DegreesOfFreedom=1
GeometryKind=chain
GeometryOptions=ConstantValues
Connectors 1 0.25

DegreesOfFreedom=1
GeometryKind=chain
GeometryOptions=ConstantValues
Connectors 1 -0.0625

Model=TjMultiOrb
```

```
potentialV 16
0 0 0 2.0 0 0 0 0
0 0 0 2.0 0 0 0 0
InfiniteLoopKeptStates=100
FiniteLoops 4
-6 100 2 6 100 2
-6 100 2 6 100 2

TargetElectronsUp=4
TargetElectronsDown=4
Threads=1

SolverOptions=CorrectionVectorTargetting,useSvd,twositedmrg,restart
CorrectionA=0
Version=version
RestartFilename=dataGS.txt

OutputFile=data_C3.txt

CorrectionVectorOmega=0.1
DynamicDmrgType=0
TSPProductOrSum=sum
CorrectionVectorFreqType=Real

CorrectionVectorEta=0.2
CorrectionVectorAlgorithm=Krylov
Orbitals=1

GsWeight=0.1

TSPSites 1 3
TSPLoops 1 1

TSPOperator=raw
RAW_MATRIX
3 3
0 0 0
0 0 0
1 0 0
FERMIONSIGN=-1
JMVALUES 1 1
AngularFactor=1
```

This input could be executed quickly on a standard laptop as

```
./dmrg -f input_Step2.inp
```

Above, the core-hole interaction strength $V_C = 2.0$ enters as a local chemical potential shift at site $c = L/2 - 1$ (see below the `potentialV` flag). We have also used a core-hole linewidth $\Gamma = 0.2$ (`CorrectionVectorEta`) and incident energy $\omega_{\rm IN} = 0.1$ (`CorrectionVectorOmega`). The solver option `CorrectionVectorTargetting` with `CorrectionVectorAlgorithm=Krylov` flag computes the $|\alpha_{c,\uparrow}\rangle$ vector in Eq. (5) of the main text using the Krylov approach. Finally, notice that the operator matrix $\hat{D}_{c,\uparrow}$ to be applied to the ground state enters directly at the end the input file. The main algorithm now bifurcates at Step 3. In fact, one needs to compute the vector $|\alpha_{j,\uparrow}\rangle$ (for some site $j = 5$, for instance) in order to get the $\Delta S = 1$ contribution to the RIXS spectrum, while $|\alpha_{j,\downarrow}\rangle$ is needed to get the $\Delta S = 0$ part. We here report only the input `input_Step3_j=5.inp` needed to get $|\alpha_{j,\uparrow}\rangle$.

```
TotalNumberOfSites=8
```

```
NumberOfTerms=4

DegreesOfFreedom=1
GeometryKind=chain
GeometryOptions=ConstantValues
Connectors 1 -0.3

DegreesOfFreedom=1
GeometryKind=chain
GeometryOptions=ConstantValues
Connectors 1 0.25

DegreesOfFreedom=1
GeometryKind=chain
GeometryOptions=ConstantValues
Connectors 1 0.25

DegreesOfFreedom=1
GeometryKind=chain
GeometryOptions=ConstantValues
Connectors 1 -0.0625

Model=TjMultiOrb

potentialV 16
0 0 0 0 0 2.0 0 0
0 0 0 0 0 2.0 0 0
InfiniteLoopKeptStates=100
FiniteLoops 4
-6 100 2 6 100 2
-6 100 2 6 100 2

TargetElectronsUp=4
TargetElectronsDown=4
Threads=1

SolverOptions=TargetingRixsStatic,useSvd,twositedmrg,restart
CorrectionA=0
Version=version
RestartFilename=data_C3.txt

OutputFile=data_C3_j5.txt

CorrectionVectorOmega=0.1
DynamicDmrgType=0
TSPProductOrSum=sum
CorrectionVectorFreqType=Real

CorrectionVectorEta=0.2
CorrectionVectorAlgorithm=Krylov
Orbitals=1

GsWeight=0.1

TSPSites 1 5
TSPLoops 1 1
```

```
TSPOperator=raw
RAW_MATRIX
3 3
0 0 0
0 0 0
1 0 0
FERMIONSIGN=-1
JMVALUES 1 1
AngularFactor=1
```

This input could be executed quickly on a standard laptop as

```
./dmrg -f input_Step3_j=5.inp
```

Above, as opposed to the Step 2 input, the core-hole interaction strength $V_C = 2.0$ enters as a local chemical potential shift at site j (see below the `potentialV` flag). As in Step 2, we have used the same core-hole linewidth $\Gamma = 0.2$ (`CorrectionVectorEta`) and incident energy $\omega_{\rm IN} = 0.1$ (`CorrectionVectorOmega`). The solver option `TargetingRixsStatic` with `CorrectionVectorAlgorithm=Krylov` flag computes the $|\alpha_{j,\uparrow}\rangle$ vector in Eq. (6) of the main text using the Krylov approach and keeps track (in the DMRG sense) of the previously calculated $|\alpha_{c,\uparrow}\rangle$ in Step 2. Even in this case, the operator matrix $\hat{D}_{j,\uparrow}$ to be applied to the ground state enters directly at the end the input file. As shown in Fig. (1) of the main text, in the Step 3 of the main algorithm, contributions coming from runs with inputs `input_Step3_j=x.inp` for all the sites `x` of the chain are needed.

Finally, we describe a generic input file needed in Step 4 to compute the real space RIXS correlator for $\Delta S = 1$. Once the vectors $|\alpha_{c,\uparrow}\rangle$ and $|\alpha_{j,\uparrow}\rangle$ are loaded restarting from Step 3, the following input `input_Step3_j=5_om=4.inp` computes the correction-vector $|x_{c,\downarrow,\uparrow}\rangle$ and, when properly executed as

```
./dmrg -f input_Step3_j=5_om=4.inp ':cddn.txt'
```

the correlator $I_{j,c}(\Omega = \omega_4) = \langle \alpha_{j,\uparrow}|\hat{D}_{j,\downarrow}|x_{c,\downarrow,\uparrow}\rangle$. Notice that the energy loss $\Omega = \omega_4$ has been computed.

Also, the option `':cddn.txt'` means that the operator $\hat{D}_{j,\downarrow}$ is being applied properly to get $I_{j,c}(\Omega = \omega_4)$. The input `input_Step3_j=5_om=4.inp` reads

```
TotalNumberOfSites=8
NumberOfTerms=4

DegreesOfFreedom=1
GeometryKind=chain
GeometryOptions=ConstantValues
Connectors 1 -0.3

DegreesOfFreedom=1
GeometryKind=chain
GeometryOptions=ConstantValues
Connectors 1 0.25

DegreesOfFreedom=1
GeometryKind=chain
GeometryOptions=ConstantValues
Connectors 1 0.25

DegreesOfFreedom=1
GeometryKind=chain
GeometryOptions=ConstantValues
Connectors 1 -0.0625

Model=TjMultiOrb
```

```
potentialV 16
0 0 0 0 0 0 0 0
0 0 0 0 0 0 0 0
InfiniteLoopKeptStates=100
FiniteLoops 4
-6 100 2 6 100 2
-6 100 2 6 100 2

TargetElectronsUp=4
TargetElectronsDown=4
Threads=1
SolverOptions=TargetingRixsDynamic,useSvd,twositedmrg,restart,minimizeDisk
CorrectionA=0
Version=version
RestartFilename=data_C3_j5.txt

OutputFile=data_C3_j5_om4.txt

CorrectionVectorOmega=0.4
DynamicDmrgType=0
TSPProductOrSum=sum
CorrectionVectorFreqType=Real

CorrectionVectorEta=0.075
CorrectionVectorAlgorithm=Krylov
Orbitals=1

GsWeight=0.1

TSPSites 1 3
TSPLoops 1 1


TSPOperator=raw
RAW_MATRIX
3 3
0 1 0
0 0 0
0 0 0
FERMIONSIGN=-1
JMVALUES 1 0
AngularFactor=1
```

The solver option `TargetingRixsDynamic` with `CorrectionVectorAlgorithm=Krylov` flag computes the $|x_{c,\downarrow,\uparrow}\rangle$ vector in Eq. (7) of the main text using the Krylov approach and by reading and keeping track of the previously calculated $|\alpha_{c,\uparrow}\rangle$ in Step 2 and $|\alpha_{j,\uparrow}\rangle$ in Step 3. Analogously to Step 3, the operator matrix $\hat{D}^{\dagger}_{c,\downarrow}$ to be applied to $|\alpha_{c,\uparrow}\rangle$ enters directly at the end the input file. We have used the same a peak broadening $\eta = 0.075$ (`CorrectionVectorEta`) and computed the correction-vector $|x_{c,\downarrow,\uparrow}\rangle$ for the energy loss $\Omega = \omega_4 = 0.4$ (`CorrectionVectorOmega`). Finally, as shown in Fig. (1) of the main text, in the Step 4 of the main algorithm, contributions coming from runs with inputs `input_Step4_j=x_om=y.inp` for all the sites `x` of the chain and for all the energy losses `y` are needed.

In the last part of this section, we introduce the input file `inputCuO.inp` for the realistic multiorbital *pd* model introduced in the main text. Below, we consider a Cu_4O_{13} cluster, with open boundary conditions and use the same parameter values introduced in the main text.

```
TotalNumberOfSites=17
NumberOfTerms=5
```

```
DegreesOfFreedom=1
GeometryKind=LongRange
GeometryOptions=none
Connectors 17 17
0 -0.75 1.5 0.75 0 0 0 0 0 0 0 0 0 0 0 0 0
-0.75 0 1.8 0 0.75 0 0 0 0 0 0 0 0 0 0 0 0
1.5 1.8 0 -1.8 -1.5 0 0 0 0 0 0 0 0 0 0 0 0
0.75 0 -1.8 0 -0.75 0 0 0 0 0 0 0 0 0 0 0 0
0 0.75 -1.5 -0.75 0 -0.75 1.5 0.75 0 0 0 0 0 0 0 0 0
0 0 0 0 -0.75 0 1.8 0 0.75 0 0 0 0 0 0 0 0
0 0 0 0 1.5 1.8 0 -1.8 -1.5 0 0 0 0 0 0 0 0
0 0 0 0 0.75 0 -1.8 0 -0.75 0 0 0 0 0 0 0 0
0 0 0 0 0 0.75 -1.5 -0.75 0 -0.75 1.5 0.75 0 0 0 0 0
0 0 0 0 0 0 0 0 -0.75 0 1.8 0 0.75 0 0 0 0
0 0 0 0 0 0 0 0 1.5 1.8 0 -1.8 -1.5 0 0 0 0
0 0 0 0 0 0 0 0 0.75 0 -1.8 0 -0.75 0 0 0 0
0 0 0 0 0 0 0 0 0 0.75 -1.5 -0.75 0 -0.75 1.5 0.75 0
0 0 0 0 0 0 0 0 0 0 0 0 -0.75 0 1.8 0 0.75
0 0 0 0 0 0 0 0 0 0 0 0 1.5 1.8 0 -1.8 -1.5
0 0 0 0 0 0 0 0 0 0 0 0 0.75 0 -1.8 0 -0.75
0 0 0 0 0 0 0 0 0 0 0 0 0 0.75 -1.5 -0.75 0

DegreesOfFreedom=1
GeometryKind=LongRange
GeometryOptions=none
Connectors 17 17
0 0 1 0 0 0 0 0 0 0 0 0 0 0 0 0 0
0 0 1 0 0 0 0 0 0 0 0 0 0 0 0 0 0
1 1 0 1 1 0 0 0 0 0 0 0 0 0 0 0 0
0 0 1 0 0 0 0 0 0 0 0 0 0 0 0 0 0
0 0 1 0 0 0 1 0 0 0 0 0 0 0 0 0 0
0 0 0 0 0 0 1 0 0 0 0 0 0 0 0 0 0
0 0 0 0 1 1 0 1 1 0 0 0 0 0 0 0 0
0 0 0 0 0 0 1 0 0 0 0 0 0 0 0 0 0
0 0 0 0 0 0 1 0 0 0 1 0 0 0 0 0 0
0 0 0 0 0 0 0 0 0 0 1 0 0 0 0 0 0
0 0 0 0 0 0 0 0 1 1 0 1 1 0 0 0 0
0 0 0 0 0 0 0 0 0 0 1 0 0 0 0 0 0
0 0 0 0 0 0 0 0 0 0 1 0 0 0 1 0 0
0 0 0 0 0 0 0 0 0 0 0 0 0 0 1 0 0
0 0 0 0 0 0 0 0 0 0 0 0 1 1 0 1 1
0 0 0 0 0 0 0 0 0 0 0 0 0 0 1 0 0
0 0 0 0 0 0 0 0 0 0 0 0 0 0 1 0 0

DegreesOfFreedom=1
GeometryKind=chain
GeometryOptions=ConstantValues
Connectors 1 0

DegreesOfFreedom=1
GeometryKind=chain
GeometryOptions=ConstantValues
Connectors 1 0

DegreesOfFreedom=1
GeometryKind=chain
```

```
GeometryOptions=ConstantValues
Connectors 1 0

Model=HubbardOneBandExtendedSuper

hubbardU 17
4 4 8 4 4 4 8 4 4 4 8 4 4 4 8 4 4

potentialV 34
3 3.5 0 3.5 3 3.5 0 3.5 3 3.5 0 3.5 3 3.5 0 3.5 3
3 3.5 0 3.5 3 3.5 0 3.5 3 3.5 0 3.5 3 3.5 0 3.5 3

InfiniteLoopKeptStates=128
FiniteLoops 5
7 1000 0
-15 1000 0 15 1000 0
-15 1000 0 15 1000 0

TargetElectronsUp=2
TargetElectronsDown=2
Threads=1
OutputFile=dataGS.txt
SolverOptions=twositedmrg,MatrixVectorKron,useSvd
Version=version
TruncationTolerance=1e-9
```

For the hopping matrix, a snake-like one dimensional geometry has been used for the cluster. Also, it is worth mentioning that the matrix

```
DegreesOfFreedom=1
GeometryKind=LongRange
GeometryOptions=none
Connectors 17 17
0 0 1 0 0 0 0 0 0 0 0 0 0 0 0 0 0
0 0 1 0 0 0 0 0 0 0 0 0 0 0 0 0 0
1 1 0 1 1 0 0 0 0 0 0 0 0 0 0 0 0
0 0 1 0 0 0 0 0 0 0 0 0 0 0 0 0 0
0 0 1 0 0 0 1 0 0 0 0 0 0 0 0 0 0
0 0 0 0 0 0 1 0 0 0 0 0 0 0 0 0 0
0 0 0 0 1 1 0 1 1 0 0 0 0 0 0 0 0
0 0 0 0 0 0 1 0 0 0 0 0 0 0 0 0 0
0 0 0 0 0 0 1 0 0 0 1 0 0 0 0 0 0
0 0 0 0 0 0 0 0 0 0 1 0 0 0 0 0 0
0 0 0 0 0 0 0 0 1 1 0 1 1 0 0 0 0
0 0 0 0 0 0 0 0 0 0 1 0 0 0 0 0 0
0 0 0 0 0 0 0 0 0 0 1 0 0 0 1 0 0
0 0 0 0 0 0 0 0 0 0 0 0 0 0 1 0 0
0 0 0 0 0 0 0 0 0 0 0 0 1 1 0 1 1
0 0 0 0 0 0 0 0 0 0 0 0 0 0 1 0 0
0 0 0 0 0 0 0 0 0 0 0 0 0 0 1 0 0
```

contains the repulsion term between the copper and oxygen orbitals, with interaction strength $U_{pd} = 1\ eV$. Finally, given the detailed discussion given above for the t-J model, inputs for the computation of the RIXS spectra at the Cu-L edge for the multiorbital pd model can be obtained straightforwardly.

* Electronic address: anocera@utk.edu
